# Longitudinal Height Growth in Children and Adolescents with Type-1 Diabetes Mellitus Compared to Controls in Pune, India

**DOI:** 10.1155/2023/8813031

**Published:** 2023-07-01

**Authors:** Sandra Aravind Areekal, Anuradha Khadilkar, Pranay Goel, Tim J. Cole

**Affiliations:** ^1^Department of Biology, Indian Institute of Science Education and Research, Pune 411008, India; ^2^Department of Growth and Endocrinology, Hirabai Cowasji Jehangir Medical Research Institute, Pune 411001, India; ^3^University College London Great Ormond Street Institute of Child Health, London WC1N 1EH, UK

## Abstract

**Background:**

Height growth is affected by longterm childhood morbidity.

**Objectives:**

To compare the growth curves of Indian children diagnosed with Type-1 diabetes mellitus (T1DM) and a control group of children without diabetes, and to see how parental height and disease severity affect the growth pattern. *Subjects and Methods*. The data came from: (i) the Sweetlings T1DM (STDM) study with 460 subjects aged 4–19 years, previously diagnosed with T1DM and followed for 2–6 (median 3) years, with repeat measurements of height and glycated hemoglobin (HbA1c), and (ii) the Pune School-Children Growth (PSCG) study with 1,470 subjects aged 4–19 years, and height measured annually for median 6 years. Height growth was modeled using SuperImposition by Translation and Rotation (SITAR), a mixed effects model which fits a cubic spline mean curve and summarizes individual growth in terms of differences in mean size, and pubertal timing and intensity.

**Results:**

SITAR explained 99% of the variance in height, the mean curves by sex showing that compared to controls, the children with diabetes were shorter (by 4/5 cm for boys/girls), with a later (by 1/6 months) and less intense (−5%/−10%) pubertal growth spurt. Adjusted for mean height, timing and intensity, the diabetic and control mean curves were very similar in shape. SITAR modeling showed that mean HbA1c peaked at 10.5% at age 15 years, 1.0% higher than earlier in childhood. Individual growth patterns were highly significantly related to parental height, age at diabetes diagnosis, diabetes duration, and mean HbA1c. Mean height was 3.4 cm more per + 1 SD midparental height, and in girls, 2 cm less per + 1 SD HbA1c.

**Conclusion:**

The results show that the physiological response to T1DM is to grow more slowly, and to delay and extend the pubertal growth spurt. The effects are dose-related, with more severe disease associated with greater growth faltering.

## 1. Introduction

Type-1 diabetes mellitus (T1DM) is a chronic disorder characterized by a deficiency in insulin production. It is the major form of diabetes diagnosed in children and is commonly referred to as “childhood-onset” diabetes. The worldwide prevalence of T1DM in children under the age of 20 years in 2021 was estimated to be 1.2 million cases (149,500 incident cases), with the highest prevalence in India [1].

Growth has previously been reported to be impaired in children with diabetes [2–4]. However, recent reports suggest that with improved diabetes control, height growth can be within the normal range [5–7]. Previous studies have used height-for-age *z*-score (HAZ) to study height growth in children with diabetes [2, 4, 8, 9]. HAZ is appropriate for analyzing height data treated cross-sectionally, where it adjusts for age and ensures that mean HAZ is relatively constant across age. However, for longitudinal data in individuals, it performs less well. The age adjustment fails to cater for individual differences in the timing of the pubertal growth spurt, so that for early maturers, HAZ rises with age and then falls again, while for late maturers, it falls then rises. These individual age trends in HAZ are both complex and hard to interpret.

A better approach with longitudinal data is to model untransformed height using, for example, the nonlinear mixed effects model SuperImposition by Translation and Rotation (SITAR) [10]. SITAR estimates (i) differential trends in height growth in two groups, for example, in children with and without diabetes, as mean differences in size, timing, and intensity [11, 12], and (ii) individual-level differences in the pattern of growth as subject-specific random effects for size, timing, and intensity [13–15]. These methods have not previously been applied to growth in children with diabetes.

Metabolic control is known to be affected by both behavioral and physiological changes during adolescence. Previous studies have not distinguished between behavioral and physiological factors influencing growth in children and adolescents with T1DM. Using SITAR, individual-level growth differences can be related to information relevant to each child's condition, such as their degree of metabolic control and diabetes duration. By characterizing individual variation in size, timing, and intensity of pubertal growth, SITAR provides a way to distinguish between physiological and behavioral influences on metabolically stressed growth.

Here we use the SITAR model to characterize height growth in children with diabetes compared to a local control population and to explore how (i) mean height growth is affected by T1DM and (ii) individual growth during puberty is related to disease severity.

## 2. Methods and Subjects

### 2.1. Datasets

The data came from two studies in Western India, namely the Sweetlings Type-1 Diabetes Mellitus (STDM) study and the Pune School-Children Growth (PSCG) study. The PSCG study here acts as a control group for the diabetes study. This study adhered to the STROBE guidelines for reporting observational studies [16].

#### 2.1.1. Sweetlings Type-1 Diabetes Mellitus (STDM)

The STDM study involved 490 children (222 boys and 268 girls) aged 1–26 years diagnosed with T1DM and visiting a tertiary healthcare center in Pune, India. T1DM was diagnosed on the basis of fasting plasma glucose >126 mg/dL or 7.0 mmol/L [17] and C-peptide <0.2 nmol/L [18]. Each subject was seen between one and six times (median 3, interquartile range 2–5) between 2013 and 2021. All the children in the study were on a basal–bolus regimen.

Height and glycated hemoglobin (HbA1c) were recorded on each occasion. Height was measured using a Seca stadiometer (Hamburg, Germany) and calibrated with standard rods, while HbA1c was measured using high-performance liquid chromatography (HPLC, BIO-RAD, Germany). Age at diabetes diagnosis was obtained from clinic records, and the mean duration of diabetes (i.e., mean age minus age at diagnosis) was calculated. Parental heights were measured at the time of the child's diagnosis, while birth weight was obtained from the birth card (where available) or parental report. Midparental height *z*-score was calculated as the mean of internally calculated height *z*-scores for mother and father. The data have been analyzed previously [19, 20].

#### 2.1.2. Pune School-Children Growth (PSCG)

The control group from the PSCG study consisted of 1,472 children (798 boys and 674 girls) aged 3–18 years recruited from three randomly selected schools catering to middle-class children from Pune, as used in a previous study [21]. Each child was seen annually, between one and six times (median 6, interquartile range 5–6) between 2007 and 2013. Measurements for children with any serious or growth-affecting illness, as assessed by pediatricians, were excluded during the initial study [21]. No further exclusion criteria were applied in the current study.

Height was measured using a portable stadiometer (Leicester Height Meter; Child Growth Foundation, London) and calibrated with standard rods. Growth curves and growth centiles based on the data have been published previously [22].

Written informed consent was obtained from parents, and verbal assent was obtained from children older than 7 years. The Ethics Committee of the Jehangir Clinical Development Centre Pune approved the data collection (dated June 26, 2007). In addition the Ethics Committee of the Indian Institute of Science Education and Research Pune approved secondary data analysis of the STDM and PSCG datasets (IECHR/Admin/2021/001). Note that the STDM and PSCG datasets differed in their design; STDM had median three measurements per child, covering a period of 2 years, whereas PSCG had median six measurements per child, covering 5 years. Thus PSCG had more longitudinal information per child, leading to a more precise mean growth curve. PSCG children were recruited from private schools, which could be a potential source of bias. However, since these schools take middle-class children, PSCG was a valid control for STDM.

### 2.2. Methods

#### 2.2.1. SITAR Model for Height Growth Curves

Height growth curves were fitted using SITAR [10]. SITAR is a mixed effects growth curve model that estimates the mean height curve as a natural cubic B-spline with degrees of freedom (*df*) chosen to optimize the fit. Subject deviations from the mean curve are captured in three subject-specific random effects: (i) *size*, which distinguishes the final height attained, (ii) *timing*, which captures the timing of puberty, and (iii) *intensity*, which provides information on the rate of pubertal growth.

The three random effects are assumed normally distributed and the residual error terms are considered to be independently and identically distributed. The fitted mean curve is also called the height distance curve, and its first derivative is the mean height velocity curve.

#### 2.2.2. Diabetes and Control Height Growth Curves

The mean curves for the diabetes and control children were estimated in two different ways: first by fitting a single SITAR model to the two datasets pooled, including fixed effects for size, timing, and intensity to distinguish between the datasets; and second by fitting separate SITAR models to each dataset. The pooled model constrains the two mean curves to be the same shape (adjusted for the fixed effect differences), whereas the separate model allows the mean curves to differ; this allows the equality of the two mean curves to be tested for.

Both ways, variants of the SITAR model were explored by considering spline curve *df* from 4 to 8. Models with log-transformed age and/or height scales and combinations of fixed effects (size and timing, size and intensity, and size, timing, and intensity) were also considered. The optimal model was the one minimizing the Bayesian information criterion (BIC) [23]. Bootstrap confidence intervals (CIs) for the mean age at peak height velocity (APV in years) and peak height velocity (PV in cm/year) were obtained using 500 resamples. The percentage of variance explained by the model was calculated as described in [24].

The optimal models fitted height versus log age, where the random and fixed effects for timing can be multiplied by 100 and viewed as percentage differences [25]. Alternatively they can be multiplied by mean APV to express them in units of months or years.

The data were analyzed using the package *sitar* (version 1.2.0.9000) [26] in the statistical language R (version 4.2.2) [27]. Prior data cleaning removed obviously errant points based on plots of height versus age (STDM: 10 points in boys and 9 in girls; PSCG: 11 points in boys and 3 in girls). The SITAR models were then fitted, and standardized residuals exceeding four in absolute value were excluded (STDM: 3 in boys; PSCG: 12 in girls). Ninty-five percent CI bands were obtained for the average SITAR curves using 500 bootstrap resamples. The mean and standard deviation by age of the bootstrapped curves were summarized as smooth curves by fitting the normal distribution family in GAMLSS (version 5.4.10) [28].

#### 2.2.3. Modelling Serial HbA1c Measurements by Age

Longitudinal trends in HbA1c were also modeled using SITAR, with the sexes pooled and a fixed effect was included to distinguish between them. The random effects for timing and intensity did not improve the fit, so the model included just the random effect for size, that is, a random intercept model.

#### 2.2.4. Relating SITAR Random Effects to Subject-Specific Covariates

The optimal SITAR height model for the STDM children was extended to explore how the three subject-specific random effects related to the following physiological covariates: midparental height *z*-score, birth weight, age at diabetes diagnosis, diabetes duration, and mean HbA1c (size random effect from Section 2.2.3). For this, the SITAR height model was extended to predict each of the three random effects as linear functions of the covariates, included as fixed effects. Subjects with no HbA1c measurements were assumed to have mean HbA1c (i.e., random intercept 0). The significance level for the analysis was set at *α* = 0.01.

## 3. Results

The datasets were cleaned and analyzed separately by sex. The age range for the study was restricted to 4–19 years since the height growth pattern in infancy is distinct from that in childhood and adolescence. The final analysis included: in STDM; 460 subjects with 1,598 height measurements and in PSCG; 1,470 subjects with 8,140 height measurements, as summarized in Table 1.

### 3.1. Average Growth Curves

#### 3.1.1. Pooled Models of Diabetes and Control Growth Curves

**Figure 1:**
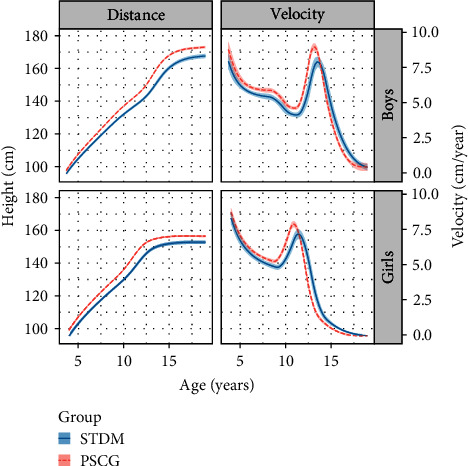
SITAR mean height growth curves and height velocity curves by sex in the diabetes (STDM) and control (PSCG) children with 95% CI bands.

Figure 1 shows the SITAR mean distance and velocity curves (95% CI) for STDM and PSCG estimated from the pooled data with 6 and 5 *df* in boys and girls respectively (variance explained 99.0% and 99.1%).  Table 2 shows the mean differences between STDM and PSCG in size, timing, and intensity. The children with diabetes were shorter than the control children, by 4.9 cm in boys and 3.8 cm in girls. Their timing of pubertal growth was also later, by 1.5 months in boys and 6.1 months in girls. In addition, their intensity of pubertal growth was lower by 9.8% in boys and 4.8% in girls.

#### 3.1.2. Separate Models of Diabetes and Control Growth Curves

SITAR height models were also fitted to each dataset separately. The optimal mean STDM curves had 6 and 5 *df* in boys and girls, respectively (variance explained 99.4% and 99.5%), while the mean PSCG curves had 6 and 4 *df* in boys and girls (variance explained 98.8% in both sexes). Mean APV and PV in the two groups are given in Table 3. Note that the values for PSCG differ slightly from before [22] due to the differing age ranges. The BIC of the pooled model was 29 units smaller than the sum of the BIC for the two separate models in boys, but 20 units greater in girls. Thus, based on BIC, the pooled model fitted better in boys but the separate model was better in girls.

**Figure 2:**
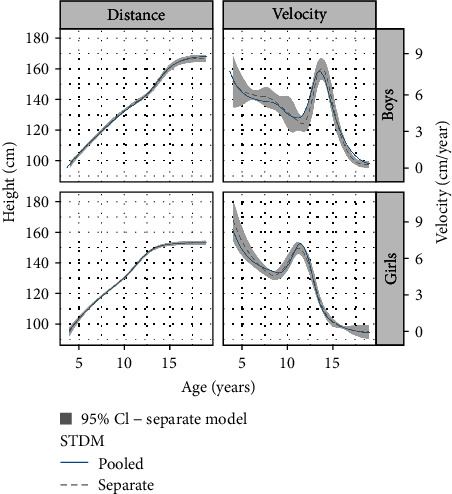
SITAR mean height growth curves and height velocity curves by sex with 95% CI bands for STDM pooled (solid blue) and separate (dotted gray).

The mean STDM distance and velocity curves estimated the two ways (with 95% CI bands for the separate models) are compared in Figure 2, with the pooled and separate models shown as solid blue and dotted gray curves, respectively. The two sets of curves are very similar in shape, showing that pooling with the control data did not materially affect the diabetes curves.

### 3.2. Modelling Longitudinal HbA1c Measurements

**Figure 3:**
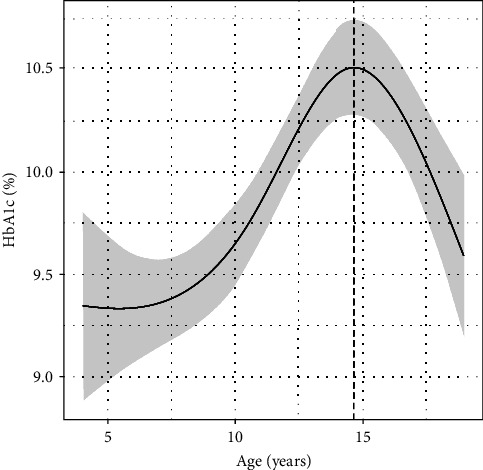
The mean HbA1c growth curve (and 95% CI band) with a peak at 10.5% at age 14.6 years.

The subject-specific HbA1c curves were summarized using SITAR with a random intercept and cubic B-spline with 3 *df* (variance explained 43%). The SD of the random intercept (mean individual HbA1c) was 0.14% and the residual SD was 0.15%.  Figure 3 shows the mean curve and 95% CI band, with HbA1c rising to 10.5% at 14.6 years and then falling again. The sexes did not differ (mean difference −0.01, 95% CI −0.04 to 0.02).

### 3.3. Individual Variation in Height Growth

We examined associations among the STDM children of the SITAR random effects size, timing, and intensity with midparental height, birth weight, age at diabetes diagnosis, diabetes duration, and mean HbA1c (the random intercept from Section 3.2). Summary statistics for the covariates are in Table 4, while the regression coefficients of the random effects on the covariates are in Table 5, where the models include all the covariates so they are mutually adjusted. Note that since birth weight proved to be unrelated to any of the random effects, it was omitted from the models.

Size was highly significantly positively associated with midparental height, age at diagnosis and diabetes duration in both sexes. Boys and girls with a 1 SD greater midparental height were, respectively, 3.3 and 3.4 cm taller. Among children who had diabetes for the same length of time, boys/girls diagnosed 1 year later were 1.1/1.1 cm taller. Among those diagnosed at the same age, those who had had diabetes 1 year longer were 0.4/0.8 cm taller. In girls, though not in boys, mean HbA1c was strongly negatively associated with mean height; a 1% higher mean HbA1c (SD 0.14%) corresponded to being 15 cm shorter, or 2 cm shorter for a 1 SD higher HbA1c.

Timing was highly significantly positively associated with both age at diagnosis and duration, with similar effects in the two sexes. Adjusted for duration, diagnosis 1 year later was associated with 4.7/3.9 months delay in age at peak velocity. Similarly, adjusted for age at diagnosis, one extra year of diabetes was associated with 5.1/4.4 months delay. Adjusted for diabetes duration, being diagnosed 1 year later corresponded to 4.8% faster pubertal growth in boys, and rather less in girls. For boys with the same age at diagnosis, one extra year of diabetes corresponded to 3.8% faster pubertal growth.

All three HbA1c coefficients were larger in girls than boys *P* < 0.03, showing the greater impact of HbA1c on growth in girls. No other coefficients in Table 5 differed significantly by sex. Parental heights were also analyzed separately; however, the coefficients for paternal and maternal height did not differ significantly for any SITAR parameters. In addition there were no significant interactions between age at diabetes diagnosis and diabetes duration.

## 4. Discussion

We studied height growth in two groups of children aged 4–19 years from Pune, India; one diagnosed with and undergoing treatment for T1DM, and the other school-children acting as controls, to explore how a metabolic disorder such as T1DM affects height growth. Historically, Indian children are reported to have poor metabolic control [8, 19, 31]. It is possible that persistent insulin deficiency despite treatment leads to impaired growth. Previous literature on growth in Indian children with T1DM, based on HAZ comparisons, has reported short stature [8] and lowered pubertal growth velocity [19]. Moreover, a 15.7% prevalence of stunting was also reported in Indian children with T1DM [32]. In this work, SITAR was used to model height and estimate an average curve for each group. Children with diabetes were comparatively shorter at all ages, and their pubertal growth was both delayed and extended compared to the control group.

SITAR assumes that height growth in individuals can be summarized by their attained height (size) and the timing and intensity of their pubertal growth spurt; hence after adjusting for these the shape of the average growth curve ought to be the same in the two groups. Growth curves in diabetes were first estimated by pooling the data to have the same underlying shape as the control group curves. Then the two sets of curves were estimated separately; this allowed the pooled and separate curves to be compared. For boys the pooled curves fitted slightly better; the opposite was true for girls. In practical terms the shapes of the diabetes curves were indistinguishable from those of the control curves after SITAR adjustment—only subtle differences were visible in the height velocity curves (Figure 2). This shows that the mechanism whereby T1DM affects growth is accurately modeled by SITAR: the underlying growth process is invariant, robustly independent of disease status. The effect of diabetes is to slow and delay growth, which leads to reduced height overall.

A SITAR model fitted to HbA1c with age shows that it rises through childhood, peaks in puberty and then falls again (Figure 3). The pubertal peak in HbA1c is likely due not only to physiological changes, such as increased insulin resistance during puberty [33], but also to behavioral resistance to lifestyle change [34]. We also analyzed how individual differences in pubertal height growth relate to child-specific physiological characteristics. We found, unsurprisingly, that children with taller parents were taller. The course of disease, in particular the age at diagnosis and the time since diagnosis, affected mean height and the timing of the growth spurt–later diagnosis and longer duration were independently associated with greater height and later puberty. However there was an important sex difference: girls were affected more in terms of size, particularly with mean HbA1c, where a 1 SD increase in HbA1c was associated with being 2 cm shorter. Boys were affected more in terms of timing and intensity, which was positively associated with age at diagnosis and the duration of the disease. Thus, broadly speaking, the impact of T1DM was to reduce height in girls, whereas in boys it slowed and delayed growth. The dependence of pubertal growth on growth hormone and testosterone in girls and boys, respectively, may be important for this observed difference as suggested by Dunger et al. [35]. A recent study by Blasetti et al. [36] also showed that shorter subjects had a higher HbA1c SDS, but they did not report any sex differences.

SITAR has previously been used in an experimental paradigm in life course epidemiology, with growth as the exposure being related to a later life outcome [11, 12]. Here we shift to a different paradigm where diabetes is the early exposure and growth in puberty is the outcome. We observe that children with diabetes grow in a different way from control children, and it is useful to put this observation into a life history framework, which deals with how individuals organize themselves in the context of scarce resources to optimize their life experiences. The transitions between different stages of growth, such as from childhood to adolescence, have been described by Hochberg and Albertsson-Wikland [37] as a period of “adaptive plasticity,” which denotes the trade-offs adopted by an organism under adverse conditions. An organism under stress, either externally (environmental) or internally (physiological), has to make the choice whether to push for fecundity, that is, to have more children, or longevity, that is, to live longer. We see that the effect of diabetes is to make the children progressively shorter through childhood, and simultaneously to delay the pubertal growth spurt and extend it. In this way the child with diabetes responds to the diabetic insult by investing fewer of its resources in growth.

A limitation of our study is the difference in design of the two datasets, with median three measurements per child in STDM compared to six measurements in PSCG. So the individual STDM growth curves were less precisely specified than those in PSCG. A reviewer has also queried the validity of the data collected during the COVID-19 lockdown. An analysis comparing results before, during and after lockdown shows that height growth was not compromised and glycaemic control was if anything marginally improved in lockdown [38]. A strength of our study is that it contrasts data on a substantial number of children with diabetes followed over time with a group of control children from broadly the same environment.

Finally, we remark that the COVID-19 pandemic signaled an urgent need for early and timely diagnosis of T1DM, flagged by studies showing a higher incidence of T1DM in children and adolescents [39] and a higher prevalence of complications during diagnosis and management such as diabetic ketoacidosis [38, 40]. Viral infections are associated with an increased risk for T1DM in genetically susceptible children [41]. These studies highlight the need for longitudinal cohorts to examine how the pandemic has affected growth in children with T1DM. As we have shown, SITAR is an excellent tool to study how diabetes affects growth.

## Figures and Tables

**Table 1:** Summary statistics for STDM and PSCG data.

Characteristic	STDM	PSCG
Subjects	460	1,470
Boys	208 (45%)	797 (54%)
Girls	252 (55%)	673 (46%)
Measurements	1,598	8,140
Boys	732 (46%)	4,455 (55%)
Girls	866 (54%)	3,685 (45%)
Age (years)	12.2 (3.8)	11.1 (3.1)
Height SDS (WHO [29, 30])	−1.24 (1.22)	−0.35 (0.97)
HbA1c (%)	9.8 (8.6, 11.3)	–

Numbers are *n* (%) or mean (SD) or median (interquartile range).

**Table 2:** Differences in the fixed effects for mean size, timing, and intensity in STDM compared to PSCG, with standard errors (SE) and *p*-values (*P*).

	Boys			Girls		
	Difference	SE	*P*	Difference	SE	*P*
Size (cm)	−4.9	0.6	0.001	−3.8	0.6	0.001
Timing (%)	1.0	0.9	0.3	4.6	0.9	0.001
Timing (months)	1.5	1.5	0.3	6.1	1.1	0.001
Intensity (%)	−9.8	1.7	0.001	−4.8	1.7	0.006

**Table 3:** Mean (95% CI) age at peak height velocity (APV) and peak velocity (PV) estimated separately for STDM and PSCG by sex.

	Boys		Girls	
	APV (years)	PV (cm/year)	APV (years)	PV (cm/year)
STDM	13.8 (13.4, 14.1)	8.1 (7.6, 8.7)	11.2 (10.7, 11.6)	6.8 (6.4, 7.2)
PSCG	13.1 (12.9, 13.2)	8.9 (8.7, 9.2)	10.9 (10.8, 11.0)	7.9 (7.8, 8.1)

**Table 4:** Summary statistics of covariates in the STDM dataset.

Variable	Boys			Girls		
	*N*	Mean	SD	*N*	Mean	SD
Maternal height (cm)	184	153.8	5.2	234	153.7	5.8
Paternal height (cm)	185	166.5	6.4	206	166.8	6.7
Midparental height (*z*-score)	185	0	1	235	0	1
Birth weight (kg)	208	2.8	1.1	252	2.7	0.6
Age at diagnosis (years)	208	8.0	4.3	249	7.9	4.0
Diabetes duration (years)	208	4.4	3.5	249	4.1	3.2

*N* is the number of subjects with measurements and SD is the sample standard deviation.

**Table 5:** Regression coefficients *β* (SE) of size, timing, and intensity on the covariates.

	Boys (*N* = 184)		Girls (*N* = 232)	
	*β* (SE)	*P*	*β* (SE)	*P*
Size (cm)				
Midparental height (*z*-score)	3.3 (0.6)	<0.001	3.4 (0.5)	<0.001
Age at diabetes diagnosis (years)	1.1 (0.2)	<0.001	1.1 (0.2)	<0.001
Diabetes duration (years)	0.4 (0.2)	0.04	0.8 (0.2)	<0.001
Mean HbA1c (%)	−8 (5)	0.1	−15 (5)	0.001
Timing (months)				
Midparental height (*z*-score)	3.4 (1.4)	0.013	0.4 (1.0)	0.7
Age at diabetes diagnosis (years)	4.7 (0.6)	<0.001	3.9 (0.4)	<0.001
Diabetes duration (years)	5.1 (0.6)	<0.001	4.4 (0.4)	<0.001
Mean HbA1c (%)	−9 (11)	0.4	−20 (9)	0.03
Intensity (%)				
Midparental height (*z*-score)	6.4 (2.2)	0.003	3.5 (2.2)	0.1
Age at diabetes diagnosis (years)	4.8 (0.8)	<0.001	1.9 (0.9)	0.04
Diabetes duration (years)	3.8 (0.9)	<0.001	2.4 (1.1)	0.03
Mean HbA1c (%)	−17 (17)	0.3	−39 (23)	0.1

*N* denotes the number of subjects included in the analysis.

## Data Availability

The data that support the findings of this study are available upon reasonable request.
